# Supplementary material for: Sex-specific cardiac remodeling in aged rats after adolescent chronic stress: associations with endocrine and metabolic factors
Source: Biol Sex Differ. 2024 Aug 23;15:65. doi: 10.1186/s13293-024-00639-7 (PMC11342553; doi:10.1186/s13293-024-00639-7)
Supplement: Supplementary file 1 — Supplementary Material 1 [file 13293_2024_639_MOESM1_ESM.docx]

**Supplemental Material**

**Title:** Sex-Specific Cardiac Remodeling in Aged Rats after Adolescent Chronic Stress: Associations with Endocrine and Metabolic Factors

**Authors:** Carley Dearing, Ella Sanford, Nicolette Olmstead, Rachel Morano, Lawson Wulsin, Brent Myers

**Endocrine Responses: psychogenic stress and metabolic stress**

Analysis of forced swim test (FST) responses showed numerous sex and stress effects (Fig. S1). Baseline corticosterone measures indicated main effects of sex [(1, 103) = 8.650, p = 0.004, η^2^ = 7.006] and stress [F(1, 103) = 4.706, p = 0.0324, η^2^ = 3.812] in young animals and a main effect of sex [F(1, 102) = 24.03, p < 0.0001, η^2^ = 17.7] in aged animals. This is maintained in the young total corticosterone response with both main effects of sex [F(1, 38) = 13.66, p = 0.0007, η^2^ = 14.78] and stress [F(1, 38) = 27.81, p < 0.0001, η^2^ = 30.09]. However, aged total corticosterone response to FST shows no significant effect. Significant sex differences were noted in both the young and aged total blood glucose response to FST with a main effect of sex [F(1, 101) = 48.31, p < 0.0001, η^2^ = 29.31] and [F(1, 102) = 67.10, p < 0.0001, η^2^ = 37.81], respectively. Interestingly, within the young mid VH group, young No CVS females mounted a smaller glucose response than both CVS females (p = 0.0253) and No CVS males (p = 0.0007). Within the high VH young animals, CVS males had a greater glucose response than CVS females (p = 0.0047). Aged animals also showed sex-specific differences with the low VH No CVS males having a greater glucose response than No CVS females (p = 0.0076) and CVS males showing a greater glucose response than their female counterparts in both the mid VH (p = 0.0019) and High VH (p = 0.0212) groups.

While glucose tolerance test (GTT) glucocorticoid responses in aged animals were impacted by VH, GTT responses in young animals showed only sex effects (Fig. S2). Young baseline corticosterone measures showed a main sex effect [F(1, 90) = 32.51, p < 0.0001, η^2^ = 24.63]. When looking at peak corticosterone levels 30 minutes following intraperitoneal injection of glucose, young animals show no significant differences. Similarly, young animals show no significant effects in total corticosterone response to metabolic challenge.


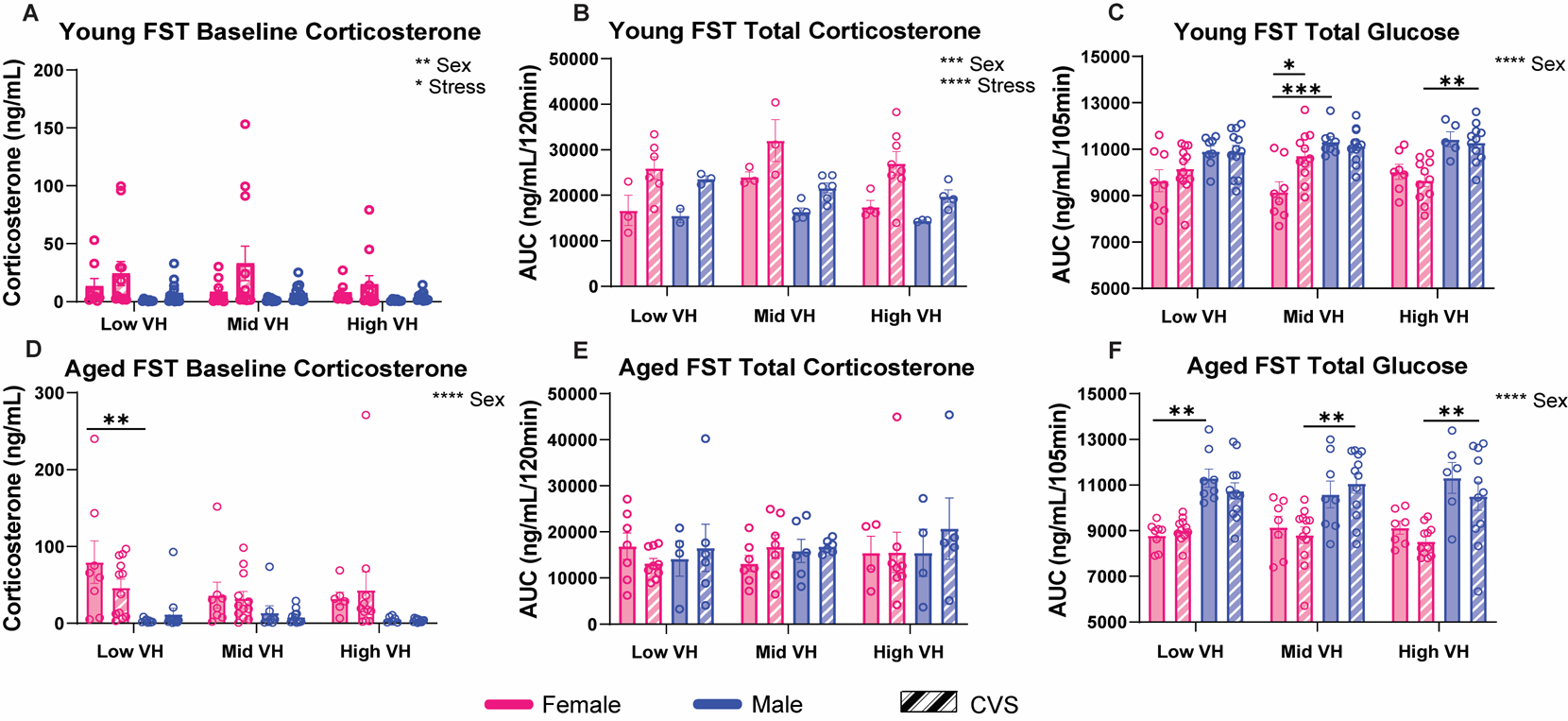


**Fig. S1.** Forced swim test (FST) endocrine analyses. Baseline plasma corticosterone was measured (A,D) two days prior to FST. Total plasma corticosterone and blood glucose were calculated from the AUC for both the young (B,C) and aged (E,F) animals. Groups were analyzed according to ventricular hypertrophy (VH) subpopulations within chronic variable stress (CVS) and sex (n = 8/sex No CVS and n = 12/sex CVS each for low, mid, and high). Data are expressed as mean ± SEM. * p < 0.05, ** p < 0.01, *** p < 0.001, **** p < 0.0001. AUC: area under the curve.


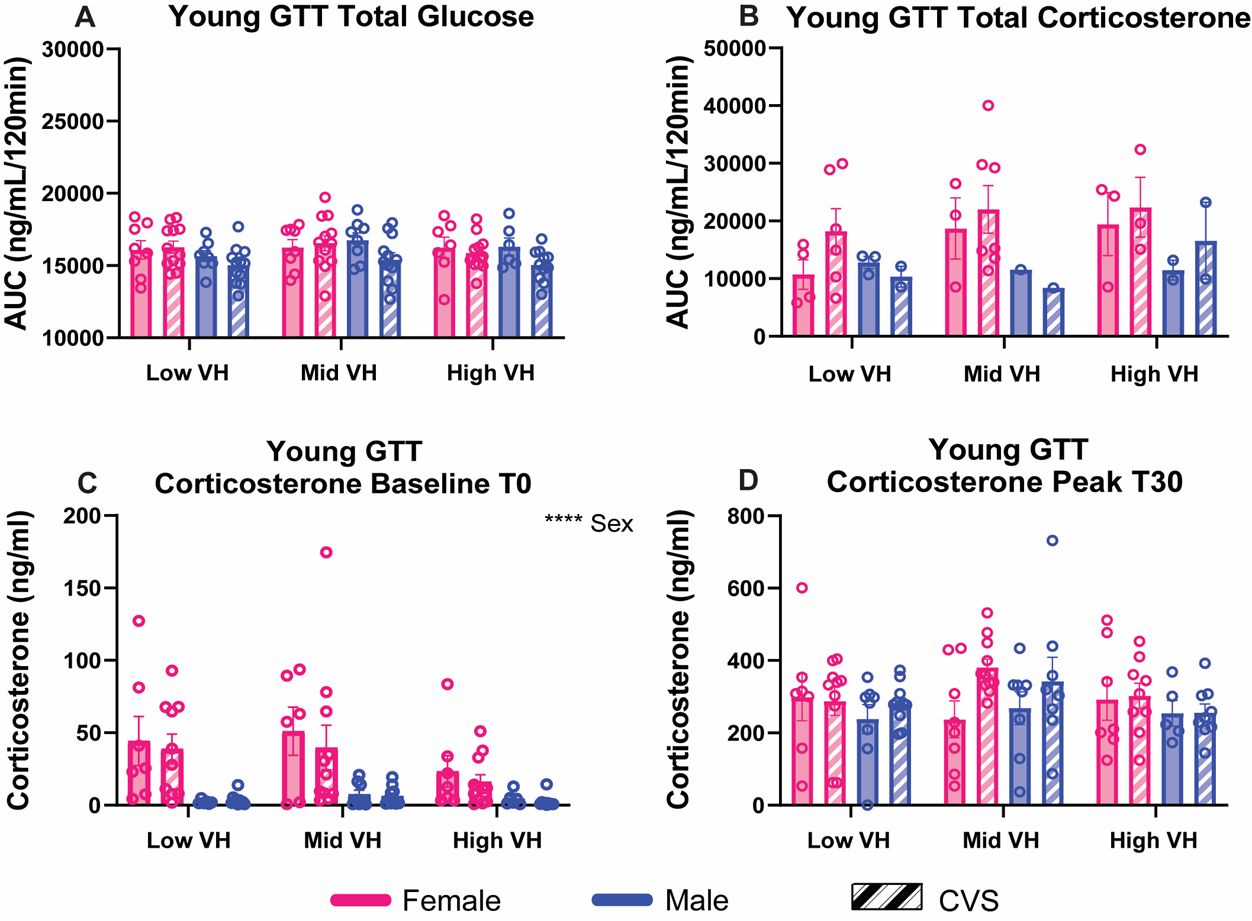


**Fig. S2.** Young glucose tolerance test (GTT) endocrine analyses. Young animals were metabolically challenged following chronic variable stress. Total blood glucose (A) and plasma corticosterone (B) were calculated from an AUC analysis. Baseline corticosterone was measured at T0 taken prior to glucose injection (C). Peak corticosterone response was measured 30 minutes following glucose injection (D). Groups were analyzed according to ventricular hypertrophy (VH) subpopulations within chronic variable stress (CVS) and sex (n = 8/sex No CVS and n = 12/sex CVS each for low, mid, and high). Data are expressed as mean ± SEM. **** p < 0.0001. AUC: area under the curve.

**Somatic Measures**

Somatic measures of body and organ weights showed sex- and stress-specific effects (Fig. S3). Measures of bodyweight-corrected spleen [F(1, 100) = 13.05, p = 0.0005, η^2^ = 10.06] and adrenal weight [F(1, 101) = 230.4, p < 0.0001, η^2^ = 66.19] showed main effects of sex. Main effects of sex were also reflected in young triglyceride levels [F(1, 101) = 84.15, p < 0.0001 , η^2^ = 38.71] and plasma cholesterol [F(1, 101) = 16.01, p = 0.0001, η^2^ = 12.36] measured immediately following CVS. Additionally, young triglycerides showed a main interaction of sex and stress [F(1, 101) = 5.10, p = 0.0261, η^2^ = 2.346]. Body weight analysis showed main effects of sex both immediately following chronic stress [F(1, 103) = 819.0, p < 0.0001, η^2^ = 84.04] and at tissue collection [F(1, 103) = 1032, p < 0.0001, η^2^ = 86.23].


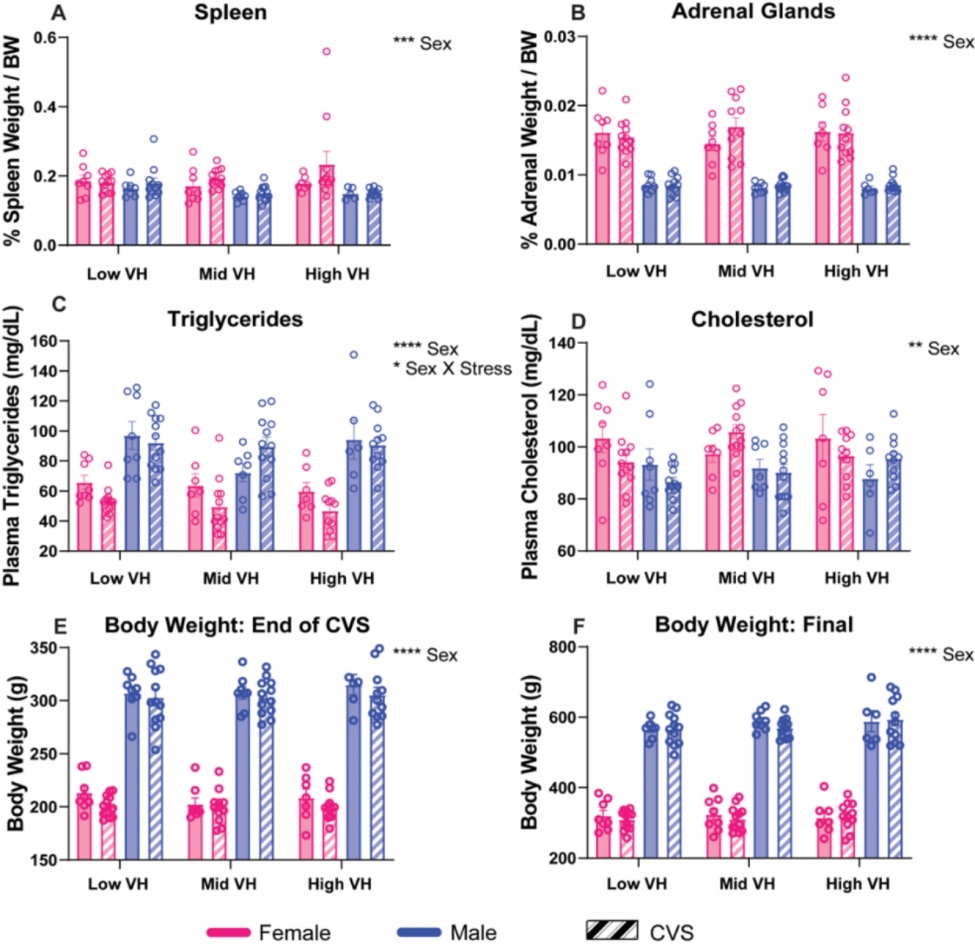


**Fig. S3.** Somatic measures following chronic variable stress. Relative spleen (A) and adrenal weight (B) were measured. Triglycerides (C) and cholesterol (D) were measured following chronic variable stress (CVS) from baseline blood samples. Body weight was measured and analyzed at both immediately following CVS and at euthanasia (F). Groups were analyzed according to ventricular hypertrophy (VH) subpopulations (n = 8/sex No CVS and n = 12/sex CVS each for low, mid, and high). Data are expressed as mean ± SEM. * p < 0.05, ** p < 0.01, *** p < 0.001, **** p < 0.0001. BW represents body weight.
